# Enhanced Drug Delivery System Using Mesenchymal Stem Cells and Membrane-Coated Nanoparticles

**DOI:** 10.3390/molecules28052130

**Published:** 2023-02-24

**Authors:** Shubham Joshi, Sarah Allabun, Stephen Ojo, Mohammed S. Alqahtani, Piyush Kumar Shukla, Mohamed Abbas, Chitapong Wechtaisong, Hussain M. Almohiy

**Affiliations:** 1 Department of Computer Engineering, SVKM’S NMIMS Mukesh Patel School of Technology Management and Engineering, Shirpur 425405, Maharashtra, India; 2 Department of Medical Education, College of Medicine, Princess Nourah bint Abdulrahman University, Riyadh 11671, Saudi Arabia; 3 Electrical and Computer Engineering, College of Engineering, Springdale, 316 Boulevard, Anderson, SC 29621, USA; 4 Radiological Sciences Department, College of Applied Medical Sciences, King Khalid University, Abha 61421, Saudi Arabia; 5 BioImaging Unit, Space Research Centre, University of Leicester, Michael Atiyah Building, Leicester LE1 7RH, UK; 6 Department of Computer Science and Engineering, University Institute of Technology, Rajiv Gandhi Proudyogiki Vishwavidyalaya (Technological University of Madhya Pradesh), Bhopal 462033, Madhya Pradesh, India; 7 Electrical Engineering Department, College of Engineering, King Khalid University, Abha 61421, Saudi Arabia; 8 Electronics and Communications Department, College of Engineering, Delta University for Science and Technology, Gamasa 35712, Egypt; 9 School of Telecommunication Engineering, Suranaree University of Technology, Nakhon Ratchasima 30000, Thailand

**Keywords:** mesenchymal stem cell (MSC), drug delivery system, pharmacodynamics, nanoparticles, pharmacokinetics, enhanced particle swarm optimization (E-PSO)

## Abstract

Mesenchymal stem cells (MSCs) have newly developed as a potential drug delivery system. MSC-based drug delivery systems (MSCs-DDS) have made significant strides in the treatment of several illnesses, as shown by a plethora of research. However, as this area of research rapidly develops, several issues with this delivery technique have emerged, most often as a result of its intrinsic limits. To increase the effectiveness and security of this system, several cutting-edge technologies are being developed concurrently. However, the advancement of MSC applicability in clinical practice is severely hampered by the absence of standardized methodologies for assessing cell safety, effectiveness, and biodistribution. In this work, the biodistribution and systemic safety of MSCs are highlighted as we assess the status of MSC-based cell therapy at this time. We also examine the underlying mechanisms of MSCs to better understand the risks of tumor initiation and propagation. Methods for MSC biodistribution are explored, as well as the pharmacokinetics and pharmacodynamics of cell therapies. We also highlight various promising technologies, such as nanotechnology, genome engineering technology, and biomimetic technology, to enhance MSC-DDS. For statistical analysis, we used analysis of variance (ANOVA), Kaplan Meier, and log-rank tests. In this work, we created a shared DDS medication distribution network using an extended enhanced optimization approach called enhanced particle swarm optimization (E-PSO). To identify the considerable untapped potential and highlight promising future research paths, we highlight the use of MSCs in gene delivery and medication, also membrane-coated MSC nanoparticles, for treatment and drug delivery.

## 1. Introduction

**Figure 1:**
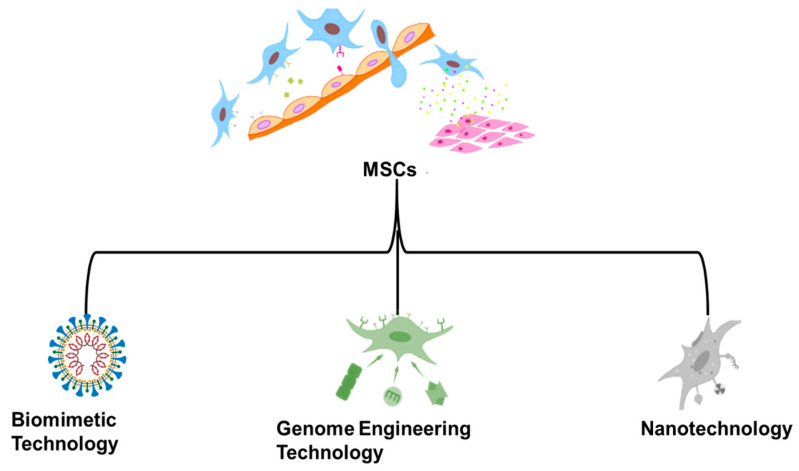
Modified-release medication delivery systems based on mesenchymal stem cells and their applications.

One of the world’s most significant new medical treatments is cell therapy. It is generally acknowledged that immune cells, somatic cells, induced Pluripotent Stem Cells (iPSCs), and stem cells may all be utilized in medical therapies. Numerous cell treatment products have already been approved for the international market [1,2,3]. As new insights are gained from biological research into everything from cell-derived peptide decorating to cell-based DDSs, the design of DDSs is constantly improving. Because of its lack of immunogenicity, intrinsic mutation rate, extended circulation duration, “lack of neurotoxicity and tumorigenicity, innate targeting capacity, and incorporation of receptors, the cell-based targeted delivery system (TDDS)” has emerged as a potential delivery technique. In addition to “RBCs, platelets, natural killer cells, neutrophils, stem cells, leukocytes, phagocytes, B lymphocytes, tumor cells, T lymphocytes, and even viruses and bacteria” have all been identified as efficient natural carriers. Some differentiated cells are difficult to collect in large enough numbers under natural settings; others cannot proliferate in vitro; and still others are difficult to manipulate. All of these factors restrict the potential therapeutic uses of this technology [4]. Many different types of tissue, such as marrow, chorion, adipocytes, cord blood, placenta, dentistry, endometrium, fetal membranes, muscle hypertrophy, lung, liver, and skin, have been used to separate MSCs for clinical research. The singular characteristics possessed by MSCs mean that they hold the potential to be used as cellular therapeutic agents in the treatment of regenerative medicine as well as immune-related conditions. [5,6,7]. For all cellular goods, safety comes first. Because of their potential to foster tumor growth, MSC products carry a certain degree of danger. Research into both possible adverse effects and the biodistribution of systemically given cells is necessary before MSCs can be widely employed in clinical practice. The effect of MSC biodistribution on tumor promotion and suppression are the subject of this review [8]. To maximize their therapeutic benefits while decreasing their hazards, MSC-DDS need to strengthen their disease-targeting capabilities. Ingenious methods have been created to enhance both the efficacy and safety of MSCs-DDS. The first part of this study provides an overarching synopsis of the most recent advancements in MSCs-DDS, followed by a brief assessment of the main challenges that prevent these therapies from being used in clinical practice [9]. Extensive discussion and examples are provided to highlight how “several illustrative skills, including nanotechnology, biomimetic technology and genome engineering technology”, have contributed to improving MSCs-DDS performance. The benefits of these advancements, which are represented in Figure 1, include but are not limited to increased drug loading capacity, improved targeted efficiency toward sick tissues, and reduced dangers related to employing live stem cells as chemotherapeutic drugs [10]. “We give a complete analysis of the use of MSCs in medicine, MSC membrane-coated nanoparticles, and gene delivery for treatment and drug administration”, to discover the vast untapped potential and highlight interesting future approaches.

Contributions of the study:

The research based on drug delivery system using mesenchymal stem cells and nanoparticles has been selected for the study. The experimental conditions used are based on the evidence from the existing literature, and significant bioactive metabolites in the effects and mechanism of action of the nanoparticles were selected. Literature published in the last seven years was selected for the proposed study.

This study primarily focuses on the biodistribution and systemic safety of MSCs to evaluate the present status of Cell therapy with MSCs.

- We investigated the mechanisms underlying the potential effects of MSCs on tumor initiation and promotion.
- In addition, the pharmacodynamics and pharmacokinetics of cell therapies, as well as the methodologies for MSC biodistribution, are discussed.
- We also highlight many other potential technologies that might be used to “advance the MSCs-DDS, comprising nanotechnology, biomimetic technology, and genome engineering technology.”
- In this study, we used analysis of variance (ANOVA), the log-rank test (LR), and Kaplan–Meier Method (KMM).
- We aim to develop a drug distribution network that utilizes E-PSO.

## 2. Related Works

In [11], the author transduced MSCs derived from murine bone marrow using lentiviral vectors, expressing IL-4 in a way that is NF-B sensitive or constitutively active (MSCs). The lipopolysaccharide (LPS) greatly enhanced the IL-4 secretion in the NF-B sensing MSCs, but the IL-4 secretion remained unchanged in the MSCs that were transfected with an upregulated IL-4 producing vector. In [12], the authors consider the history of cell treatments, concentrating on the benefits of stem cell therapy. This article will present cell sheet technology as a promising new cell treatment that has already shown therapeutic promise for regenerating various organ tissues, including the heart, liver, and kidneys in a variety of clinical trials. The potential and predicted therapeutic effects of cell sheet technology using MSCs are reviewed. MSCs were better able to take in and maintain nanoparticles inside the cell after they were TAT-functionalized. Additionally, nanoengineering MSCs did not change their ability to migrate or differentiate. The authors therefore showed that MSCs modified with TAT-functionalized particles are potent carriers for the targeted delivery of anticancer medicines to tumors, significantly boosting the therapeutic effectiveness of these medications [13]. “In this study, a chemical compound that is advantageous for bone regeneration was created by coupling Simvastatin (Sim), a novel treatment alternative for bone regeneration, with “Graphene Oxide (GO)”, a technique that has newly earned significant attention as a DDS.” [14]. Cytotoxicity tests were used to examine the impact of modifying GO with polyethylene mine to establish a steady and homogeneous compound with Sim. In [15], transforming growth factor b3 (TGF-b3) was adsorbed on graphene oxide (GO) flakes that were subsequently integrated into a keratin gel. The chondrogenic differentiation of human MSCs was also evaluated after they were enclosed in the same gel. Research has shown that GO flakes absorbed > 99% TGF-b3 with a release of 1.7%. Adsorbed TGF-b3 maintained the same shape as the soluble form (free protein) but showed improved structural strength. The stem cell membrane disguised targeted delivery mechanism in cancers in this review. To this end, the authors discussed the processes through which stem cells might “home in” on malignancies. Methods for modifying membranes and preparing nanoparticles (NPs) that have been coated with membranes from stem cells were then summarized. NP coatings on stem cell membranes have the potential to achieve tumor targeting, improved biocompatibility, and efficient drug loading [16]. In [17], the authors discussed the creation of MSC exosomes as a drug carrier, as well as the techniques of and benefits associated with exosome drug administration. The benefits and drawbacks of employing exosomes as medication delivery vehicles were discussed. Considering the present pandemic situation, exosomes are an intriguing target for innovative nano intervention, and the authors quickly outlined the latest findings and progress regarding exosomes in their study. The current review will focus on the molecular significance of exosomes in the fight against severe lung pathological diseases. Researchers have also examined the nano design of exosomes for improved utility, stability, and storage with the goal of establishing exosomes as a ready-to-use therapeutic [18]. The authors in that study used MSC loaded with anticancer NP as drug delivery vehicles to specifically target lung cancer. Drug uptake efficiency by MSC was shown to be greater than that of fibroblasts. The majority of MSC were also found to be trapped in the lungs of both rabbits and monkeys [19]. Mice with tumors were utilized to trace the movement of MSCs, which were also employed to transport drugs. “Lentiviral particles were used to transduce anaplastic thyroid carcinoma (CAL-62) cells, and human breast cancer (MDA-MB-231), resulting in the expression of mCherry and Renilla luciferase (mCherry-Rluc) reporter genes” [20]. Despite recent encouraging findings from earlier studies that supported the utilization of the MSC-secretome in IMIDs, research on humans using the Masters Administration program is still in its infancy. Ultrasound contrast is an important medical imaging tool for measuring the stability of urinary bladder pressure over time. Ultrasound contrast is created by injecting a solution of micron-sized bubbles into the bloodstream, which are then detected by ultrasound scanners. The contrast can measure the changes in bladder pressure in real time and can be used to diagnose and monitor several conditions, such as bladder outlet obstruction and neurogenic bladder [21]. Results from [22] summarize the research on the molecular mechanism of MSC-secretive in immunoassays and offer interpretations of these positive physiological activities. In [23], an injectable “hyaluronic acid-tyramine (HA-TA)” hydrogel loaded with cells was supplemented with “platelet lysate (PL)”, an autologous and low-cost source of growth factors. The researchers examined how platelet lysate affected hMSCs’ capacity to adhere to and differentiate into chondrocytes.

MSCs have recently been studied as potential cellular therapies for various diseases, including cancer. MSCs have been shown to have antitumor effects in preclinical studies, such as, inhibition of tumor growth, induction of tumor cell apoptosis, and modulation of the immune system to overwhelm tumor growth. Therefore, the utilization of MSCs in cancer therapy is still in its early stages. Several clinical researches have shown the biodistribution and safety of MSCs for tumor therapy. In these studies, MSCs were shown to be safe to administer, and that the MSCs migrated to the tumor site and persisted for several weeks. However, the systemic safety of MSCs is unclear. Previous studies have reported increased levels of cytokines in the blood following MSC administration, suggesting potential systemic effects. Further investigation is necessary to comprehend the systemic safety of MSC-based cell Therapy [21].

MSCs have been found to have many applications in preclinical and clinical research. These cells can distinguish into various types of cell and might be used to generate tissue-specific cells for regenerative medicine and cancer therapy. MSCs have also been shown to have antitumor properties and can be used to target and kill cancer cells. MSCs can be engineered to produce cytokines and other molecules that can inhibit tumor metastasis and growth. Additionally, MSCs may be utilized for delivery drugs or therapeutic agents directly to the tumor site, providing a more targeted approach for cancer treatment. MSCs can also stimulate and modulate the immune system to combat cancer. They can be used to deliver immunomodulatory molecules such as cytokines or chemokines to the tumor site. This can help activate the immune system and increase the responsiveness to tumor cells [24].

MSCs are multipotent cells with the capability to distinguish into numerous cell types. They can be used in various regenerative and therapeutic treatments, and their responses to different environmental conditions must be carefully evaluated. Depending on the method of cultivation, such as in two-dimensional (2D) cell suspensions or three-dimensional (3D) hydrogels, different responses can be observed [14].

In a 2D environment, MSCs typically form a monolayer of cells. This environment allows for ease of analysis and manipulation, and a homogeneous environment can be easily established. Although they can be grown in a well-defined and controlled environment, MSCs in 2D cell cultures may not exhibit normal morphologies or physiological responses. For example, the expression of certain genes may be upregulated or downregulated in 2D compared to 3D. In contrast, 3D environments resemble the in vivo microenvironment more closely. MSCs can form aggregates of cells that can interact with both each other and the extracellular matrix [11].

Targeted drug delivery using MSCs is a satisfactory approach for the dealing with various diseases. This technique involves the utilization of mesenchymal stem cells to deliver drugs directly to cells that are responsible for the disease. The mechanism of activation for this type of drug delivery is highly specific and involves the release of various proteins, molecules, and other factors that interact with target cells.

The first step in the mechanism of activation is the binding of the drug to mesenchymal stem cells. This is achieved by attaching a ligand, such as an antibody, to the drug molecule. The drug molecule then binds to stem cells, allowing the drug to be delivered to the target area. The second step in the activation mechanism is the release of the drug from stem cells. This release occurs through a process called transduction, which involves the use of various signaling molecules, such as cytokines, to initiate the drug’s release. Once released, the drug binds to the target cells, allowing localized and effective delivery of the drug. Finally, the third step in the mechanism of activation is the expression of the drug in target cells [14,15].

This study [23] compiles the most up-to-date information available on genetically engineered MSCs, which may co-transduce many therapeutic molecules, including suicide genes, to exert powerful ant carcinogenic activities against the development of a wide range of malignancies. MSCs secrete exosomes, which are tiny vesicles. These exosomes have been highlighted because of their potential to assist as a unique therapeutic platform for the targeted administration of a wide variety of therapeutic medicines. The EMA decided to include all “structures” with sizes of less than 1000 nm which are constructed to have special characteristics, that can enhance site-specific drug delivery and drastically change toxicological profiles, spurring the EMA to incorporate all nanomedicines because they commonly have a wider size range than the proposed definition. This allows for case-by-case assessment. Nevertheless, proper application of this law should need proven analytical techniques for nanoparticle characterization, which are not now available [21]. This is in spite of that there are many guidelines for the measurement methods of chemical parameters in these kind of matrices.

Even if the safety of several substances has been established, it is important to look at the in vivo behavior and toxicological profile as nanoparticle complexity increases due to the inclusion of ligands, the utilization of synthetic formulations, or the application of coatings. Direct cell toxicity, hemolytic effects, long-term accumulation, nanoparticle aggregation, and/or immunogenic activities are the main safety concerns. The distribution, metabolism, and excretion of nanoparticles in vivo are all factors that go into their toxicological assessment [24]. Table 2 summarizes the work done in tabular form.

## 3. Statistical Analysis

In this study, we employed ANOVA to examine the data statistically. The survival of untreated and treated cells was compared using the Kaplan–Meier and log-rank tests. All statistical tests were conducted with a significance threshold of * *p* < 0.05.

### 3.1. ANOVA

Analysis of variance, or ANOVA, is a statistical procedure that can be used to extract relevant subsets of data from large-scale reports of variability. When there are three or more data sets, “a one-way ANOVA” is performed to investigate the relationship between the variables. Average sums of squares in the null model based on the anthropocentric principle are used to calculate the classic analysis of variance (ANOVA) F-statistic. Using a least-squares method in which all variances are held constant, the parameters are determined. It might be stated as:

(1) $$N=\frac{MG_{between}}{MG_{error}}$$

where

(2) $$MG_{between}=\frac{\sum_{j=1}^{i}o_{j}{\left({\underline{P}}_{j}-\underline{P}\right)}^{2}}{i-1}$$

and

(3) $$MG_{error}=\frac{\sum_{j=1}^{i}\sum_{r=1}^{q_{j}}{\left(P_{jr}-{\underline{P}}_{j}\right)}^{2}}{z-i}$$

The formula for the Welch test statistic is:

(4) $$y=\frac{\sum_{j=1}^{i}y_{j}\left[\frac{{\left({\underline{P}}_{j}-\tilde{P}\right)}^{2}}{\left(i-1\right)}\right]}{1+\frac{2\left(i-2\right)}{i^{2}-1}\sum_{j=1}^{i}\left[\frac{{\left(1-\frac{c_{j}}{y}\right)}^{2}}{\left(z_{j}-1\right)}\right]}$$

where $C_{i}$ = $\frac{m}{d_{1}^{2}}$, *v* = $\sum_{j=1}^{i}X_{i}$ and $P=\frac{1}{Y}\sum_{j=1}^{o}C_{j}V_{j}$ is defined as:

(5) $$m=\frac{i^{2}-1}{3\sum_{j=1}^{q}\left[\frac{{\left(1-\frac{C_{i}}{y}\right)}^{2}}{\left(z_{ji}-1\right)}\right]}$$

To define the Brown–Forsythe test statistic, we say:

(6) $$M^{*}=\frac{\sum_{j=1}^{q}h_{j}{\left({\underline{P}}_{i}-P\right)}^{2}}{\sum_{j=1}^{q}\left(\frac{1-h_{i}}{z}\right)G_{j}^{2}}$$

If Lo exists, then M* should be distributed according to a centered *M* distributed with o–1 degrees of independence and m, whereby m is specified to be

(7) $$\frac{1}{n}=\frac{\sum_{j=1}^{q}t_{j}^{2}}{\left(z_{j}-1\right),t_{j}=\frac{\left(\frac{1-z_{j}}{z}\right)G_{j}^{2}}{\sum_{j=1}^{q}\left(\frac{1-z_{j}}{z}\right)G_{j}^{2}}}$$

The generalized *p*-value is now calculated as B = 1–l, where k is the sample size, yielding the generalized b-value.

(8) $$f=Z\left(J_{q-1,z-q}\left(\frac{z-q}{q-1}{\tilde{d}}_{x\;}\left(\frac{z_{1}d_{1}^{2}}{X_{1}X_{2},\ldots ,X_{q-1}},\frac{z_{2}d_{2}^{2}}{X_{1}X_{2},\ldots ,X_{q-1}},\frac{z_{3}d_{3}^{2}}{\left(1-X_{2}\right)X_{3},\ldots ,X_{q-1}},\ldots \frac{z_{1}d_{o}^{2}}{\left(1-X_{o-1}\right)}\right)\right)\right)$$

In an F-distribution with independent variables, a forecast is made for the independent Beta random variable using $J_{q-1,z-q}$

(9) $$X_{r}\sim Beta\left(\overset{r}{\underset{j=1}{\sum }}\frac{\left(z_{j}-1\right)}{2},\frac{z_{r+1}-1}{2}\right),r=1,2,\ldots ,o-1$$

To determine the significance level, we integrate the expected value numerically in the *p*-value calculation for Beta stochastic process.

### 3.2. Kaplan–Meier (KM) Method

In a randomized controlled trial (RCT) with right-censoring, patient-level data is utilized to calculate the probability of experiencing the time to event t, $G^{RN}\left(t\right)$ using the Kaplan–Meier (KM) technique. The approach is effective because it reduces the IPD to a sequence of intervals of time $\left[0,d_{1}\right),\left[d_{1},d_{2}\right)\ldots ..,\left[d_{1},\infty \right)$. We have structured them in this way to ensure that there is always something happening at the beginning of each interval. Kaplan–Meier estimates include the number of events that happen at the start of each time period *m* = 1, 2, where rcm is the number of people who have been censored on the interval, and nm is the number of patients who were still at risk well before the increment began.

(10) $$m_{n+1}=m_{n}-t_{n}-x_{n}$$

Kaplan–Meier estimates of survival functions need the statistics provided by the Kaplan–Meier data $G^{RN}$ ($t_{m}$) at event time $t_{m}$:

(11) $$G^{RN}\left(dn\right)=\overset{n}{\underset{i=1}{\prod }}\frac{n_{i}-t_{i}}{m_{i}}={G^{RN}}_{\left(d_{n-1}\right)*\frac{m_{n}-t_{n}}{m_{n}}}$$

(12) m=1, 2, …, r

“Let $T_{ji}$. is the failure time, where j=1,2,…,n index (the patients), and $i=1,2,\ldots ,K_{j}$ index (within the *i*th patient’s dental implants). $\delta_{ji}$ indicates the repressing indication”.

(13) $$\begin{array}{c} \overset{n}{\underset{j=1}{\sum }}\overset{R_{j}}{\underset{i=1}{\sum }}\overset{R_{j}}{\underset{f=1}{\sum }}\left[\frac{J\left(T_{ji}\leq d\right)\delta_{ji}}{V_{m}\left(D_{ji}\right)}\overset{n}{\underset{j^{\prime }=1}{\sum }}\overset{R_{j}}{\underset{i^{\prime }=1}{\sum }}\frac{J(D_{j^{\prime }i}\leq min\left(D_{ji^{\prime }}d\right)\delta_{ji{}^{\prime }}}{{\left(V_{m}\left(D_{j{}^{\prime }i{}^{\prime }}\right)\right)}^{2}}\right][\frac{J\left(T_{ji}\leq d\right)\delta_{ji}}{V_{m}\left(D_{ji}\right)}\\ -\overset{n}{\underset{j^{\prime }=1}{\sum }}\overset{R_{j}}{\underset{i^{\prime }=1}{\sum }}\frac{J(D_{j^{\prime }i}\leq min\left(D_{ji^{\prime }}d\right)\delta_{ji{}^{\prime }}}{{\left(V_{m}\left(D_{j{}^{\prime }i{}^{\prime }}\right)\right)}^{2}}] \end{array}$$

where

(14) $$V_{m}\left(d\right)=\overset{n}{\underset{j=1}{\sum }}\overset{R_{j}}{\underset{i=1}{\sum }}J\left(D_{ji}\geq d\right)$$

For regular cases, $n=m,\;R_{j}=1$, since each patient only has one implant, all data is independently collected. This variance formula reduces to Greenwood’s variance formula.

(15) $$\overset{n}{\underset{j=1}{\sum }}\frac{J\left(D_{j}\leq D\right)\delta_{j}}{V_{m}\left(D_{j}\right)}-\overset{n}{\underset{j^{\prime }=1}{\sum }}\frac{J(D_{j^{\prime }i}\leq min\left(D_{ji^{\prime }}d\right)\delta_{ji{}^{\prime }}}{{\left(V_{m}\left(D_{j{}^{\prime }i{}^{\prime }}\right)\right)}^{2}}$$

where

(16) $$V_{m}\left(d\right)=\overset{n}{\underset{j=1}{\sum }}\overset{R_{j}}{\underset{i=1}{\sum }}J\left(D_{j}\geq d\right)$$

### 3.3. Log-Rank Test

The log-rank test compares two groups based on the difference in their anticipated number of occurrences, i.e., *E*_1_ and *E*_2_ where 0_1_ and 0_2_ represent the overall number of observed occurrences in every group, respectively. The test statistic is:

(17) $$Log-rank\;test\;statistic=\frac{\left(0_{1}-E_{1}\right)}{E_{1}}+\frac{\left(0_{2}-E_{2}\right)}{E_{2}}$$

The projected number of things at the time of each incident in each group is added together to estimate the total number of incidents for the two groups as a whole. Multiplying the risk of an event occurring at a particular time by the number of people in a given group who were still surviving at the commencement of that timeframe yields the expected number of random occurrences at that moment for those groups.

## 4. MSC-Based Mechanisms of Action

Some ways in which MSCs could utilize their useful effects have been hypothesized. Previous research suggested that MSCs may travel to injury sites, where they would either develop into fully functioning cells or merge with injured cells to restore the injured tissue. Recent research has shown that MSC effects may be mediated in part by paracrine factors, mitochondrial transfer, and extracellular vesicle secretion.

### 4.1. Paracrine Effects

Paracrine substances, such as chemokines, cytokines, microRNAs, and growth factors, are secreted by MSCs. Paracrine factors released by MSCs after transplantation or injection of isolated secreted factors may promote a return to a normal microenvironment in injured tissues and hasten their recovery. Paracrine factors released by MSCs have significant effects in the areas of immunomodulation, tissue regeneration and repair, antifibrosis, anti-apoptosis, and angiogenesis. This has led to several studies aimed at optimizing growth conditions for MSCs to guide their secretome toward medicinal substances. Changes have been made by using MSCs from various tissues, adjusting oxygen levels, incubating with growth factors or pretreating with cytokines, increasing the number of passages, growing MSCs in three dimensions (3D), and applying mechanical strain. Their immunomodulatory properties make MSCs a promising therapeutic strategy for inflammatory diseases, including MS, graft-versus-host disease, systemic lupus erythematosus, Crohn’s disease, and type 1 diabetes. “In an inflammatory microenvironment, proinflammatory cytokines, such as IL-1β, IL-23, IL-6, TNF-α and IFN-γ, can stimulate MSCs to secrete anti-inflammatory factors for example nitric oxide (NO), TNFα stimulated gene (TSG)-6, galectins, transforming growth factor (TGF)-β, prostaglandin E2 (PGE2), and IL-10”. By causing macrophage polarization to shift toward an M2 phenotype, paracrine chemicals generated by MSCs have been shown to inhibit both innate and adaptive immune responses. Cell growth, angiogenesis, and death are all aided by substances secreted by MSCs. The growth and angiogenesis-promoting substances that MSCs may release include, for example, “basic fibroblast growth factor (bFGF), insulin-like growth factor (IGF), TGF-β, vascular endothelial growth factor (VEGF), secreted frizzled-related protein-1/2 (SFRP1/2), stromal cell-derived factor (SDF)-1α, angiopoietins, and vascular endothelial growth factor (VEGF)”. The function of tumor-promoting factors is shown in Table 1.

### 4.2. Mitochondrial Transfer

Mitochondrial malfunction is characteristic of aging and has been linked to the origin of many illnesses. Therefore, mitochondrial transfer using MSCs as a source has shown promise as a therapeutic technique, since it may restore or replace dysfunctional mitochondria in specific sick cells. There is evidence that, in inflammatory or hypoxic conditions, mitochondrial transfer from MSCs to injured epithelial or endothelial cells enhances the formation of TNT and gap junctions and guards against apoptosis in the recipient cells. A method of mitochondrial transfer through TNT by which iPSC-derived MSCs could diminish alveolar injury and fibrosis has also been discovered. There is some evidence that MSCs’ source tissue affects their capacity to transport mitochondria. In some research, transfer of mitochondria happened in both directions, which was shown to boost leukemic cell resistance to chemotherapeutic drugs. When chemotherapeutic drugs induced oxidative stress after being cultivated in vitro, “T cell acute lymphoblastic leukemia (T-ALL) cells relocated their mitochondria to the BMMSCs while getting relatively some mitochondrial from the BMMSCs, leading to improved chemo resistance in the T-ALL cells”. By inhibiting mitochondrial extracellular transport and the cellular adhesion molecule ICAM-1, T-ALL cells were once again sensitized to the chemotherapy medication. The plasma membranes are fundamentally similar to other cell organelles’ membranes, including those of the mitochondria, nucleus, endoplasmic reticulum, Golgi apparatus, and lysosomes. Drug carriers with cell organelle membrane coatings may benefit from increased therapeutic effectiveness. For instance, covering nanoparticles with nuclear, mitochondrial, or lysosomal membranes may avoid the downstream cancer therapy pathways’ resistance to anticancer drugs. Nanoparticles with nuclear membrane coatings may increase the effectiveness of transfection during gene therapy. According to research, nanoparticles with a coating on the mitochondrial membrane have the ability to selectively bind ligands to the mitochondrial membrane and neutralize poisons. Recent studies have begun to use cell organelle membranes to modify the surfaces of nanoparticles. Future research will provide new treatment possibilities because of the enormous potential of cell organelle membranes. According to investigations using scanning electron microscopy and Fourier transform infrared spectroscopy, nanoparticles were able to enter the stratum corneum. With the help of tight junction proteins and keratin, the nanoparticles might improve the transdermal effectiveness of isotretinoin, according to Western blotting. Additionally, nanoparticles improved endocytosis, boosting medication penetration and skin absorption. The literature examining the enhanced drug delivery system using mesenchymal stem is shown Table 2.

### 4.3. Extracellular Vesicle (EV) Transfer

The use of MSC-derived extracellular vehicles (EVs), a non-cellular treatment option for MSC-based therapy, is becoming more popular. This method avoids the potential drawback of lineage differentiation when used in the wrong context. A cell may release a variety of EVs when it is stimulated or dies, including membrane-enclosed exosomes, micro vesicles, and apoptotic bodies. Vesicles appear in a variety of shapes and sizes, and they include a vast variety of substances, such as proteins, miRNAs, and mRNAs. Although their involvement in MSC-mediated cellular treatment is still unknown, it is already accepted that MSCs are essential for a range of biological processes and for promoting cell-to-cell communication. The beneficial benefits of MSC exosomes have been seen in animal models of a variety of diseases and injuries, including autoimmune uveitis, macular degeneration, heart attack, “wound healing, type 1 diabetes, bone regeneration, burn harm, a traumatic brain injury, and spinal cord injury”. It has been shown that the generation of inflammatory cytokines in immune cells, such as interferon type 1 beta, interleukin 1 beta, tumor necrosis factor-alpha, interleukin 6, and macrophage inflammatory protein 1, is inhibited by exosomes generated by MSCs. Additionally, in an animal model of type 1 diabetes, exosomes generated from MSCs dramatically elevated levels of the anti-inflammatory cytokines TGF-β, IL-4, and IL-10. Exosomes generated from mesenchymal stem cells were shown to increase local production of the liver-regenerating cytokines, transforming growth factor beta, and hepatocyte growth factor in a drug-induced liver damage model. “Exosomes secreted by MSCs play a crucial role in regulating wound healing and tissue repair by activating STAT3, ERK, and Akt pathways and inducing the production of HGF, IGF1, NGF, SDF1, and TGF-β. The injected MSC-derived EVs not only traveled to and accumulated in the damaged tissue, but also in the lung, liver, and spleen”.

### 4.4. Enhanced Particle Swarm Optimization

For drug delivery system optimization, enhanced particle swarm optimization (E-PSO) is applied. Utilizing the attributes of the drug delivery system optimization using MSE allocation model, a unique PSO approach is created. For each particle in the PSO technique, a matrix of T rows and *M* columns may be constructed, $M=\sum_{j}j\;and\;t=\sum_{i}i$. The particle coding is as follows:

(18) $$particle=r_{11}\;r_{12}\;\cdots \;r_{1M}\;r_{21}\;r_{22}\;\cdots \;r_{2M}\;\cdots \;\cdots \;\cdots \;\cdots \;r_{t1}\;r_{s2}\;\cdots \;r_{tM}$$

where *t* represents the overall number of sources and m represents the number of sectors. The distribution across different sectors is shown using two-dimensional arrays. However, the addition of the identical column in a vertical direction shows the actual total amount allocated within a certain department. The pseudocode for E-PSO is represented by Algorithm 1.

The BSO algorithm for the allocation model for optimizing includes the following phase:

Phase 1: Let the algorithm’s fitness function match the overall goal function, or

(19) $$E\left(R\right)=\left[\overset{}{\underset{i=1}{\sum }}\overset{}{\underset{j=1}{\sum }}\left(d_{ji}-a_{ji}\right)r_{ji}\beta_{ji}\right]\times \lambda_{1}+\left|\overset{}{\underset{i=1}{\sum }}C_{i}-R\right|\times \lambda_{2}+\left[\overset{}{\underset{I}{\sum }}0.01\;RO_{0}O_{i}g_{i}\right]\times \lambda_{3}$$

Phase 2: Initialize the model’s parameters in step two. According to the optimization, the inertia factor, population size m, learning factors d1 and d2, and the highest allowed repeat frequency S are all calculated. Randomly produced particles are assembled in a group. Each starting particle’s Oj is set to the value of the moment.

Phase 3: Determine each particle’s fitness individually.

Phase 4: The individual ideal position pi is compared with the fitness determined in phase 3. If it is superior, pi is substituted with the value. Otherwise, nothing is altered.

Phase 5: The individual ideal position Oj and the global optimal position oh are compared in step 5. oh is substituted if it is superior. After that, Equation is used to update it (19).

Phase 6: If it is less than S, go on to Step 3; otherwise, stop.
**Algorithm 1: Pseudocode for E-PSO***E-PSO (* *while generation_lev(1)< generation_lev(1)_max do* *if m <= n then* *Initialize Swarm_lev(m)* *while generation_lev(m)< generation_lev(m)_max do* *for each particle do* *Particle move* *while m < n do* *m++* *E-PSO()* *Evaluation* *Best update* *generation_lev(m) ++* *if m > 1 then* *fitness(particle_lev(m − 1)) gbest(swarm_lev(m))* *Dispose Swarm_lev(m)* *m--*

## 5. Tumor Initiation, Promotion, and Suppression as a Potential Risk of MSCs

Considering MSCs’ excellent immune modulation capacity and the lack of tumor initiation risk, cell therapy including MSCs is a viable therapeutic method. However, there is ongoing worry that MSCs may foster a dangerous expansion of tumor cells. MSCs have the capacity to differentiate in a manner similar to fibroblast cells, which can differentiate in order to form “cancer-associated fibroblasts (CAFs)” in tumor settings. The tumor microenvironment must include regional fibroblasts, endothelial cells, immune cells, and mesenchymal stem cells (MSCs). According to a growing body of research, the tumor niche is not just trophic for cancer cells but also closely linked to the initiation and growth of tumors. It may also enhance characteristics linked to cancer stemness, such as cells’ capacity to invade, migrate, and withstand chemotherapy. Using MSCs from varying tissue sources, culture methods, and cancer types may provide conflicting findings and interpretations. However, when their propensity to migrate to tumors is exploited, MSCs may act as therapeutic carriers, transporting anticancer drugs directly to the locations where they will be most effective. “MSC-related drug carriers might deliver fresh expectation for cancer treatments, especially for late-stage tumors, which represent a substantial health burden but for which there are no curative medicines”.

## 6. FND-Based Cell Tracking Tool for In Vivo Therapy

Successful cell treatment relies on close in vivo monitoring of transplanted cells. Investigational new drug (IND) applications for cellular products need pharmacological studies to establish not only the manufacturer’s safety but also its biodistribution after transplanting in animal models, as well as its pharmacokinetics (PK) and pharmacodynamics (PD). For cell therapy to be successful, three major challenges must be overcome: (i) whether therapeutic cells lose their efficacy after being transplanted, (ii) what doses are effective in healing illnesses, and (iii) what composition and dosage method ensures that the medicine reaches its intended target.

**Figure 2:**
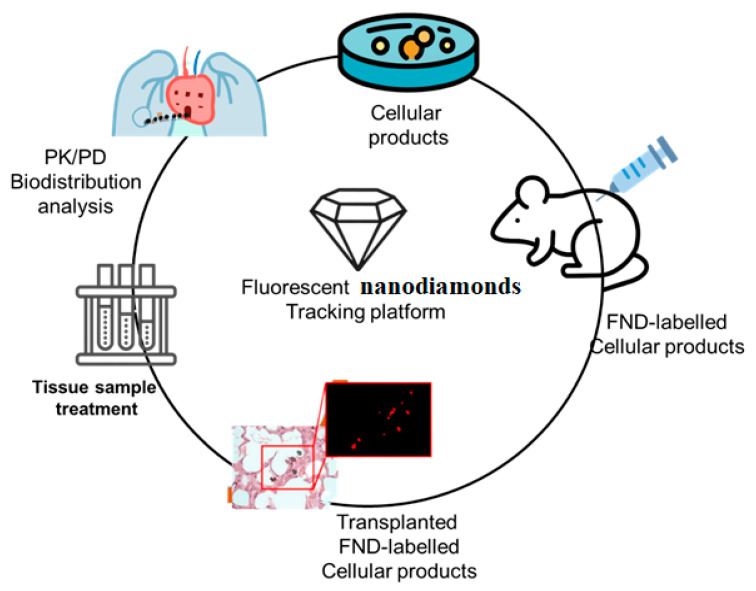
Procedures for the labeling and tracking of pcMSCs with fluorescent nano diamonds (FNDs) and an examination of their bio distribution.

**Figure 3:**
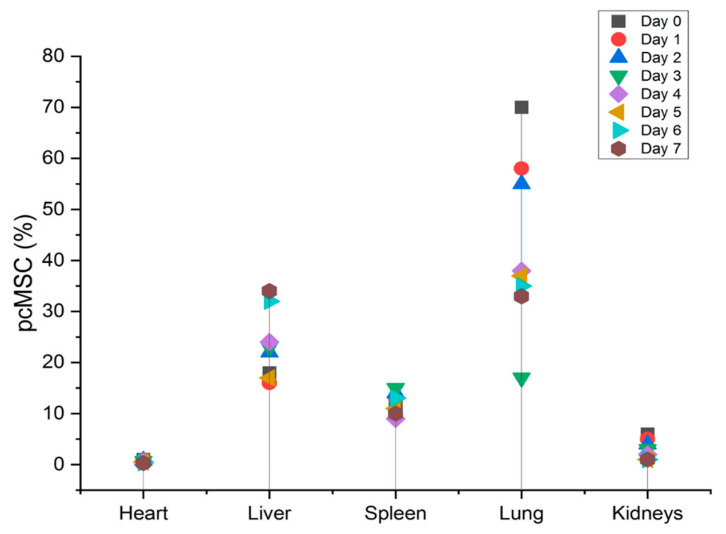
Location of FND-labeled pcMSCs in apparently healthy mice.

New cell-based therapies, or cell therapy, have emerged in the last decade, challenging the conventional wisdom that medicine could only work with organic substances of low molecular weight or with massive macromolecules. Like other drugs, knowledge of the pharmacology of cell-therapy product is necessary for these products to be used effectively in healthcare situations. For example, the sample selection process involved in tissue slice and PCR makes it tough to deliver PD and PK information for the entire animal, which prohibits these tests from providing significant information on in vivo cell behavior. However, a novel approach to high-bandwidth entire-organs therapy and evaluation is provided by the FND-labeled tracking systems, which is essential for determining the cellular therapy’s pharmacokinetics (PK), pharmacodynamics (PD), and biodistribution, as depicted in Figure 2. After obtaining histological sections from the animal, this technique not only offers quick and extremely accurate cell localization data but also allows for a straightforward analysis of any type of animal tissue in only one tube. The validation and analysis phases of this process are quicker and more efficient than those of the qPCR sampling approach, and the data it produces are more reliable. With the help of a normal mouse model, we demonstrated that the FND-labeled platform has the potential to give confirmation of cell biodistribution. One week after birth, FND-labeled placental choriodecidual lattice mesenchymal stem cells (pcMSCs) were analyzed for their biodistribution in a mouse model, as shown in Figure 3.

The use of FNDs for labeling and tracking of MSCs has been studied in both in vivo and in vitro models. In vitro studies have found that FNDs can be used to accurately track and monitor the behavior of MSCs. For example, FNDs have been used to identify the migration patterns of MSCs, helping researchers to better understand the role of these cells in regeneration and tissue repair.

FND-labeled MSCs are powerful tools for tracking the location of cells in live animal studies. This is because nanodiamonds are nontoxic and their fluorescent properties can be easily detected in their environment. Recently, the location of fluorescent nanodiamonds-labeled MSCs was studied in apparently healthy mice. The study found that the majority of labeled MSCs were found in the lungs of mice. Specifically, the labeled MSCs were located mainly in the alveolar septa and interstitial spaces. Furthermore, a significant proportion of MSCs was found in the spleen and liver of the mice. In addition, labeled MSCs were present in the kidneys, small intestine, and bone marrow. These findings suggest that fluorescent ND-labeled MSCs be able to effectively utilised to track the location of cells in live animal studies. These results also suggest that MSCs may have the capability to transfer to different organs and tissues in the body. Further studies are required to better understand the mechanisms underlying this phenomenon.

Upon intravenous treatment, up to “70% of FND-labeled pcMSCs were found in the lungs, which is in line with the pulmonary first-pass effect”. As pcMSCs are bigger than the micro capillaries of the lung, the lack of available space can cause them to become trapped. Over time, after intravenous injection of FND-labeled pcMSCs, researchers found that these cells left the lungs and moved to other organs or sites of injury. However, in both the heart and also the kidneys, the frequency of FND-labeled pcMSCs declined.

**Figure 4:**
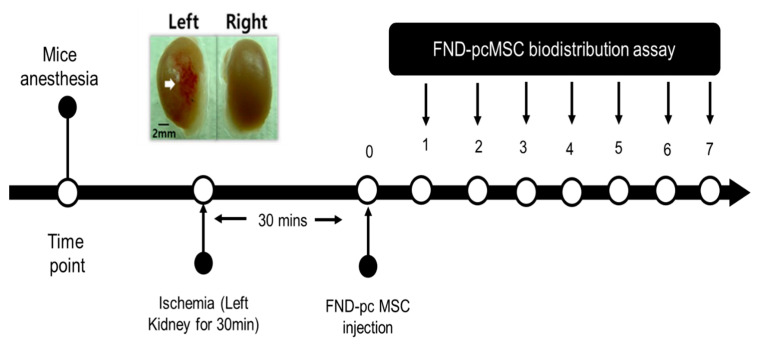
Analysis of the biodistribution of pcMSCs labeled with fluorescent nanodiamonds (FNDs) in a mouse model of kidney ischemia–reperfusion injury.

**Figure 5:**
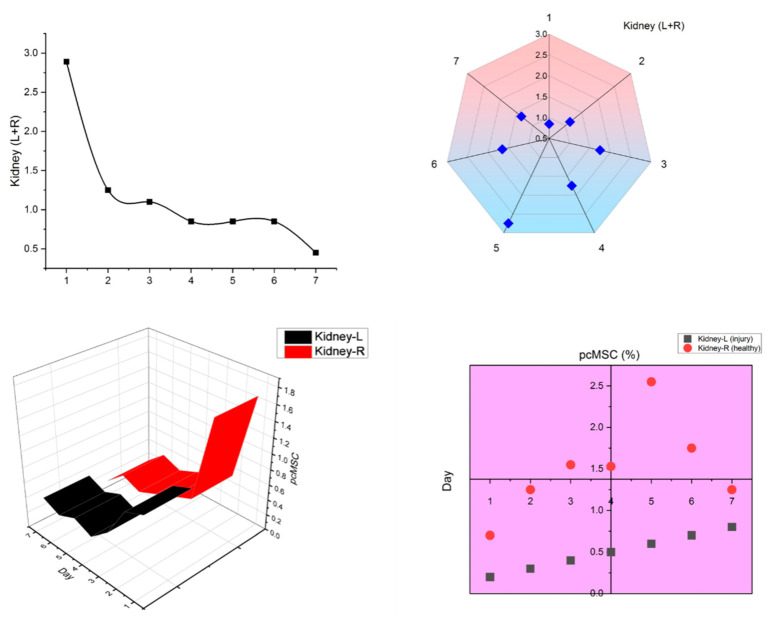
Biodistribution of FND-labeled pcMSCs in the healthy kidney mouse model (red); Biodistribution of FND-labeled pcMSCs in ischemia–reperfusion kidney injury mouse model (black).

Since it has been shown that MSCs will migrate to damage sites, we created a model in which the left kidney suffered from ischemia and subsequent reperfusion, depicted in Figure 4, and tested the hypothesis that MSCs would migrate to damaged areas by observing whether FND-labeled pcMSCs injected into the portal vein would show up in the injured kidney. Kidney-healthy mouse model showed a gradual decline in pcMSC numbers in both kidneys, as shown in Figure 5. Conversely, on day 5 (3%) after injury, the highest number of FND-labeled pcMSCs were seen in the mouse model’s injured kidney. As can be observed in the bottom panel there were many more pcMSCs that FND-labeled MSCs in the damaged kidney (L kidney) than there were in the healthy kidney (R kidney). In the normal right kidney, the percentage of pcMSCs that were labeled with FND was stable throughout time (0.25%). (R kidney) (*P) Based on these findings, it seems that kidneys can redistribute MSCs in vivo, and that the proportion of MSCs that migrate to kidneys is about 4%. In addition to its speed and precision, this method also poses no danger to the cells being studied. The cell’s survival, proliferation, differentiation, and immunomodulatory are all maintained during the FND-labelling process, giving it a very biocompatible technology.

MSCs have been identified as potential tools for tracking pharmacokinetic and pharmacodynamics processes. Pharmacokinetics are the processes of drug absorption, distribution, metabolism, and elimination from the body. MSCs provide a unique method to study these two processes because they can be tracked over an extended period. MSCs tracking methods can be used to differentiate between pharmacokinetics and pharmacodynamics. These approaches include tracking the movement of cells in a tissue or organ as well as the effects of drugs on various cells. This can help researchers understand how a drug is absorbed and distributed throughout the body, as well as how the body responds to the drug. In addition, tracking approaches can help researchers identify the metabolic pathways of drugs and determine how the drugs are eliminated from the body.

## 7. Technologies for Improving MSCs-DDS

Several methods have been tested to increase the effectiveness of MSCs-DDS despite the difficulties that have been identified. Nanotechnology and targeted gene editing are used to modify MSCs for increased medication loading capacity and homing efficiency. Recent studies have shown that biomimetic technologies may significantly improve mitochondrial DDS by providing advantages, such as high drug loading rates and equivalent or better absorption capabilities compared to wireless providers without the hazards caused by employing live cells. Here, we will describe the many ways in which these technologies have been used to improve MSCs-DDS [8,9,10,11]. MSCs can be used in regenerative medicine and in the therapy for a variety of illnesses. In order to make the best use of this potential, it is important to develop technologies that can improve the efficiency of MSCs. One technology developed for this purpose is gene editing. This method involves the use of the CRISPR-Cas9 technology to introduce genetic changes into MSCs. This technology can be used to modify MSCs so that they become more effective for tissue regeneration and more disease resistant. Other technologies for improving MSCs include cell engineering and therapy. Cell engineering involves manipulating cells to induce desirable characteristics, such as increasing their capacity to differentiate into different cell types [10,11]. Cell therapy comprises the use of MSCs to supplant damaged or lost cells in the body that result in muscle degeneration or heart failure.

### 7.1. Improvements Using Nanotechnology

To improve the therapeutic capability of MSCs-DDS, researchers have begun to experiment with several different functional or drug-loading NPs. Fluorescence imaging revealed that MSCs had taken up two types of NPS—lipid nano-capsules containing coumarin-6 dye and polylactic acid NPs—which gave them the capacity to migrate toward glioblastoma. These results specify that MSCs might serve as a delivery mechanism for drug-loaded NPs in the context of tumor targeting, creating a flexible multicomponent delivery platform that integrates MSC homing with the high loading capacity of multistage nano-vectors (MSVs). This synergy resulted in a fivefold increase in loading capacity without impairing cellular processes in any way. Both passive transport and active endocytosis play a role in the absorption of NPs. The biomedical materials and targeted drug delivery using nanoparticles is shown Table 3.

NPs to MSCs’ surfaces by chemical crosslinking or by physical connections mediated by hydrophilic and electrostatic connections might improve the rate at which they load drugs. An innovative technique of attaching NPs to the cell’s surfaces as drug stores for distribution to damaged areas uses a polymer known as a “cellular backpack” that may be applied as patches on cell surfaces. The effectiveness of MSCs as drug delivery vehicles may be compromised, however, by prolonged exposure to NPs, which can alter their properties. Several researchers have considered the impact of NPs loaded with MSCs. Another common nanomaterial for drug loading is carbon nanoparticles (NPs), which have a cytotoxic effect on human MSCs since they disrupt the expression of genes involved in cell death. NPs not only increase the targeting effectiveness of MSCs-DDS, they also boost the ability of MSCs to load drugs. With the use of nanotechnology, we can see the movement and dispersion of MSCs-DDS. More research is needed to assuage the worries about the safety and stability of MSCs-DDS which have arisen due to interactions between NPs and MSCs.

### 7.2. Improvements Using Genome Engineering Technology

Delivering foreign genes into host cells based on their sequence specificity is the basis of genome engineering technology. This robust groundwork for gene editing in mammalian cells has been laid by the rapid growth and detection of cutting-edge gene-editing tools most recently. Genome engineering has been employed to boost MSCs-targeting DDS’s efficiency by adding some functional genes. Genome engineering techniques may also improve MSCs-efficacy DDSs in pathological conditions. Genetic modification of miRNA-378, for instance, improves MSC survival in a hypothermic environment compared to unmodified MSCs, which might lead to better drug delivery efficiency and therapeutic outcomes in the face of peroxidation. By including the stanniocalcin 2 gene in their programming process, MSCs may be better able to withstand peroxidation and live longer. To maximize the efficacy of MSCs-DDS, their genetic makeup is often modified by the use of viral or nonviral gene vectors to overexpress targeted proteins. There has been a significant amount of success with viral-based approaches to genetic engineering for MSCs. The most extensively researched viral gene vectors currently are retroviruses, lentiviruses, and adenoviruses. The transduced gene may become permanently incorporated into the host cell’s genome after infection with a lentivirus or retrovirus, allowing for steady expression. However, this kind of gene integration might lead to insertional mutagenesis and oncogene activation. Genetic modification of MSCs is often accomplished via the use of nonintegrated viral vectors, such as adenovirus, which are not harmful to people and may impart long-term gene expression. Although this approach reduces some of the dangers of gene integration, the question of whether adenoviruses are immunogenic remains unanswered. A considerable percentage of the human population has developed antibodies against adenovirus, which significantly lowers the virus’s in vivo potency. Several research groups, concerned about the dangers of viral transduction, have instead concentrated on nonviral vectors as a viable alternative method of delivering genes.

### 7.3. Improvements Using Biomimetic Technology

Several new and present researches have demonstrated that MSCs risk supporting the advancement of tumors and their metastasis, which somewhat mitigates the promising potentials of MSCs for targeted medication delivery. There is also the possibility that MSC pharmacokinetics, pharmacodynamics, or vital properties may be altered as a result of interactions between live cells and loaded substances. Immune privilege and biocompatibility are only two examples of qualities that may be affected by modifications to the structure of the cell surface and the identity of molecular structures deposited on the barrier. Recently, the idea of using MSC membrane biomimicry as medication transporters has been presented. By using a hypotonic solution and gentle homogenization, researchers were able to remove the cytoplasm from MSCs, resulting in ‘ghost’ cells; these cells were then sonicated to form nanosized carriers, which they dubbed ‘nano hosts’ (NGs). In addition, physical coelution is often used in the manufacture of membrane-coated NP. Through this process, NGs can preserve the complex structure required for efficient biocompatibility, covering the membrane composition and the useful chemicals discovered on the surfaces of ghost cells. This biomimetic approach allows NGs to take on the properties of biological membranes. Proteins involved in cell motility, intercellular communication, and immunoregulation are preserved using this method, ensuring that not only physical–chemical features but also distinct biological activities are maintained. NGs’ innate targeting capability, minimal immunogenicity, and clearance by blood-filtering organs make them suitable candidates for targeted drug administration. One common study shows that NGs produced from MSCs may block prostate cancer in a mouse model by retaining their tumor-targeting ability even after being loaded with a ligand that induces death through the tumor necrosis factor pathway. Biocompatible and effective drug delivery was further proven by showing that NGs made from MSCs could successfully eradicate nude mouse model human prostate tumor cells. In comparison to living cells, NGs excel at quality assurance and have the advantage of being permanent carriers for drugs or genetic material. For instance, the consequences of lung entrapment on distribution effectiveness from intravenous injection are mitigated by NGs’ controllable size, which is frequently smaller than stem cells. With their biological features and the potential for functional integration with synthetic nanomaterials, biomimetic platforms in drug delivery have an exciting future. The next version of DDS, which was developed from MSCs-DDS, was also mentioned in connection with this platform. Hybrid cell membrane systems have been fabricated using a several different types of cells, with typical cells include erythrocytes, stem cells, immunological cells, and even cancer cells.

The emergence of 3D bio printing technology has revolutionized the field of cell therapy by allowing precise engineering of tissue and organ constructs. This technology is based on 3D printing techniques and includes the layer-by-layer deposition of cells and materials to create 3D scaffolds for tissue regeneration. Using this technology, researchers can create complex tissue architectures with high degrees of control and uniformity. This precision has enabled the development of new cell therapy methods, such as tissue engineering and stem cell therapy. Stem cell therapy is a type of cell therapy that uses stem cells to repair or replace damaged tissues or organs. By utilizing 3D bio printing technology, researchers can create 3D scaffolds that can be populated with stem cells, allowing for the precise placement of cells within the scaffold. This precision has enabled the development of effective stem cell therapies tailored to individual patients [15,25].

Hybrid systems, which are made by combining the membranes of different cells, have a wider variety of membrane molecules than those of single-cell membrane systems, and thus confer more desirable bio functions, including increased retention time, specificity in targeting inflammation, and immunity evasion. This was empirically shown in research that utilized a functionalized drug carrier made of membrane hybrids produced from erythrocytes and platelets. Increased medication functionality, such as increased detoxification (from RBC membrane) and targeted delivery to tumors, was proven by the authors, highlighting the benefits of the membrane hybrid system (from platelet membrane). As an alternative to employing cell membranes alone, a new method has been developed that involves infusing the membranes with liposomes at optimal ratios to generate a more stable drug carrier with favorable release profiles while maintaining the homing potential. Keeping the NPs’ cell membrane coating intact is a significant obstacle. However, given the technology currently available, it is hard to either guarantee that all NPs are uniformly coated without cell membranes or to extract all lattice NPs from the cell preparations. Multiple cutting-edge strategies have been created to improve this process.

**Figure 6:**
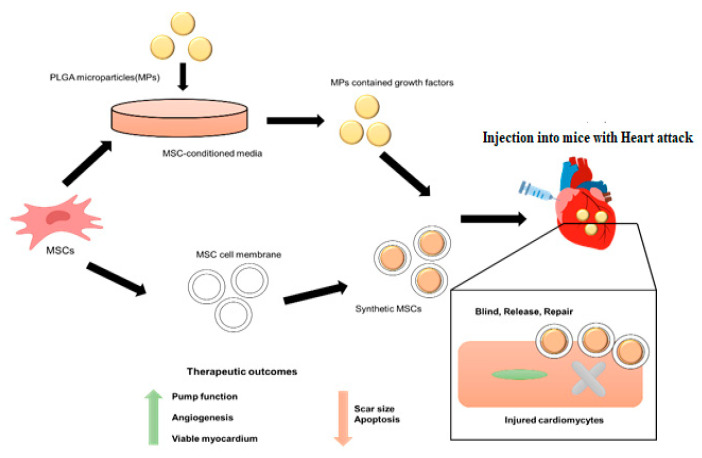
To encourage tissue recovery through cell proliferation, remuscularization, and angiogenesis, synthetic MSCs release growth factors that have been loaded.

Significant buildup at the tumor site and reduced reticuloendothelial system (RES) clearance were seen when photodynamic treatment (PDT) was combined with the MSCs membrane. First, a tiny solid crystal nucleus was created, and this was followed by nuclear maturation as the cell expanded. Physical extrusion was used to cover the particles with a membrane, mimicking the structure of a stem cell so that light could be converted to electricity (Figure 6).

## 8. Therapeutic Uses of MSCs in Cell Therapy: Potency and Safety

Recent and future decades hold much hope for MSC treatment because of its promising potential. The patient’s well-being must always come first in the development of any cutting-edge medical technology [15]. Despite the promising results shown in preclinical investigations, including the potential to control the immunological milieu and stimulate tissue regeneration, the possibility of tumor development or promotion remains a major worry with this therapy. Different immunological profiles of MSCs may promote tumor growth, depending on where they came from or how long they were cultured. In addition, due to the double-edged sword nature of MSCs’ potent immune modulation potential, it is crucial to assess not only the unique qualities of MSCs but also the patient’s immunological status before, during, and after therapy [17]. Artificial engineering may reduce tumor induction and enhance tumor-suppressing activity of mesenchymal stem cells, according to some research. The use of genetically engineered MSCs also carries additional safety hazards. Several drug trials have shown the stability of MSC-treated patients, although most of these trials have so far only examined safety relatively briefly and have not evaluated malignant cell indicators. Research on the safety of systemically injected MSCs was recently reexamined using a meta-analysis that re-evaluated 55 randomized controlled studies that included over 2000 participants [3]. Patients who were given MSCs were far more likely to develop a fever. Patients treated with MSCs had a considerably decreased chance of mortality, and they did not face an increased risk of infection, ectopic tissue development, malignancy, or thrombo-embolic events. Every design for clinical research should include a strategy for proactively tracking potential dangers, such as instant allergic reactions, local problems, vascular roadblocks, and systemic hazards [4,19].

MSCs are promising candidates for delivery of therapeutic agents. However, there are certain limitations when loading MSCs with therapeutics. For instance, some therapeutic agents, such as small molecules, cannot be directly loaded onto MSCs because they are too small to adhere to the cell surface. Furthermore, the loading capacity of MSCs is restricted by the size and number of receptors on their surfaces. The most common approach for loading therapeutic agents onto MSCs is to engineer cells to express specific receptors that can bind to the therapeutic agent. This approach can be used to target various types of therapeutic agents, such as peptides, proteins, antibodies, and even DNA. Additionally, therapeutic agents can be loaded via endocytosis, adsorption, cell-penetrating peptides, and other methods. The types of therapeutic agents that can be targeted using MSCs include drugs, gene therapy agents, cell-based therapies, immunomodulatory, and nanomaterials [6,8].

MSC-DDS methods offer many advantages over targeting and loading particles with the same therapeutic agents. For example, MSC-DDS methods provide a more efficient and less invasive delivery of therapeutic agents directly to the target spot, allowing for a higher concentration of drugs to be delivered. MSC-DDS technology can also be engineered to deliver a wide range of drug molecules with higher precision and efficacy. Additionally, targeting and loading particles with the same therapeutic agents have drawbacks, such as loss of control over the drug release profiles due to the lack of specificity of the drug delivery mechanism. However, MSC-DDS methods can achieve higher drug concentrations because of their ability to target specific tissues and release drugs in a controlled manner. This is particularly advantageous for drug delivery because it allows for more precise targeting and sustained release of therapeutic agents. Typically, MSC-DDS methods can achieve drug concentrations up to 10–100 times higher than those of other targeted drug delivery systems. This allows higher doses of drugs to be delivered at the target site, leading to better efficacy and improved patient outcomes [17,19].

In addition, MSC therapy should be carefully evaluated for individuals with a history of ischemia disorders, such as “cardiovascular diseases, lung fibrosis, concurrent neoplasm, or a family history of hereditary cancer”. The infusion method, pace, and cell dosage must be recorded. “Preclinical data from cell and animal investigations, as well as transcriptome, epigenome, proteome data, cell populations, possible potency biomarkers, and MSC product profiles from a variety of tissues and production procedures, are required”. Phase 1 and 2 trials have so far provided insufficient data to conclude MSCs’ therapeutic effectiveness across a wide range of illness indications. Important parameters impacting MSC potency in vivo include, as we have highlighted in this study, the range of cell growth settings and the different MSC tissue sources. It is therefore necessary to create surrogate potency tests in a preclinical animal model. Cell therapy based on MSC has considerable promise for the therapy of persistent inflammation-related chronic inflammatory diseases. In translational applications, the novel inflammatory inducible mechanism may lessen side effects and thereby increase treatment effectiveness and prognosis [11]. The clinical use case and implementation plan for MSC sheet treatments are based on clinical trial involvement employing autologous, patient-derived cell sheets in seven disease categories, and this preclinical evidence is expected to continue to grow [12].

There is still room for improvement in MSC precision treatment, which may be seen in the absence of fully developed methods for measuring the efficacy of cell pharmacology, cell distribution route, and cell-drug interaction. The efficacy of in vitro assays to detect MSC potency is still up for debate, even though matrix tests have been defined as a platform for determining the indicators for MSC strength. Mixed lymphocyte response (MLR) assays, which use allogeneic human peripheral blood mononuclear cells, are a frequent example of an experiment that can test the immunomodulation potential of MSCs. However, difficulties in robustness, accuracy, and repeatability raise serious concerns. There is also a need for further research on the link between in vitro tests and in vivo preclinical/clinical data. Whether MSCs retain their efficacy after being frozen as the so-called “cryo stun effect,” which occurs when MSCs are frozen, has been shown to reduce their therapeutic effectiveness, which might explain why certain clinical studies with MSCs have failed. Moreover, an acclimatization period or IFN licensing before cryopreservation may restore, in whole or in part, the MSCs’ survival and function. In conclusion, future MSC product release criteria should include the utilization of standardized potency testing. Thus, it is important to emphasize the creation of surrogate potency tests for various illness indications. Optimal cryopreservation and thawing procedures might be another element that needs further research.

## 9. Pharmacological Activities and Clinical Applications of MSCs and Membrane Coated Nanoparticles

MSCs and MCNs are two of the most important developments in pharmacological activity and clinical applications. MSCs are multipotent stem cells which can distinguish into specialized cells and can be used in regenerative medicine and tissue engineering. They can be used to treat several different diseases, such as diabetes, Parkinson’s disease, heart disease. MCNs are tiny particles composed of a thin layer of lipids that are used to deliver drugs and medications directly to target sites [19].

Membrane-coated nanoparticles have recently been investigated as promising drug delivery systems for tumor therapy. The use of MCNs allows the delivery of a wide range of bioactive metabolites to the target site, providing an effective way to maximize therapeutic efficacy. These metabolites have been shown to affect tumors in various ways, including regulation of cell proliferation and apoptosis, promotion of angiogenesis, and modulation of immune responses. In addition, the membrane-coated nanoparticle coating acts as a barrier to the absorption of foreign molecules, preventing their accumulation and thus helping to avoid unwanted side effects [1]. The most significant bioactive metabolites identified for use with membrane-coated nanoparticles are polysaccharides, proteins, and lipids. Polysaccharides such as chitosan and hyaluronic acid are capable of binding to tumor cells and delivering therapeutic agents to the target site. Proteins, such as monoclonal antibodies, can be used to target specific receptors on tumor cells, allowing for greater precision in drug delivery. Lipids, such as phospholipids, can act as effective delivery vehicles for lipophilic drugs [8].

Membrane-coated nanoparticles have various pharmacological activities, including immunosuppressive, antitumor and anti-inflammatory activities. Along with targeting tumors, they can be used to reduce inflammation, promote tissue healing, reduce pain, promote wound healing, and treat autoimmune diseases. Membrane-coated nanoparticles have been used in various clinical applications. They can be used to deliver drugs, vaccines, and imaging agents directly to a target site. They can also be used both to deliver stem cells to a target site to enhance therapeutic effects and to deliver genetic material for gene therapy [20].

MSCs are a unique type of stem cell that have the capability to distinguishes into a range of tissues, such as bone, fat, muscle, and cartilage. They also possess the ability to produce various cytokines and growth factors that can provide anti-inflammatory, immunomodulatory, and regenerative activities. Recent studies have shown that MSCs can be used to treat various diseases, including cancer. MSCs can be used to deliver anticancer agents, such as chemotherapeutic drugs, to cancer cells, resulting in enhanced antitumor effects. In addition, MSCs can be used in immunotherapy, where they stimulate the immune system to fight cancer. Membrane-coated nanoparticles are a promising platform for tumor therapy. These particles are composed of a biodegradable polymer matrix with a thin membrane layer that can encapsulate and deliver a wide range of therapeutic agents. Membrane-coated nanoparticles have the advantages of sustained drug release, high drug loading, and improved drug stability [24].

## 10. Discussion

The growth of safe and efficient DDSs is of the utmost importance for effective disease treatment. Current drug delivery systems pose several challenges in terms of targeting, safety, and efficacy. In recent times, there has been a surge of interest in the utilization of MSCs and membrane-coated nanoparticles as promising drug delivery systems to improve disease management [3,4]. Latest researches have explored the potential of MSCs and membrane-coated nanoparticles for enhanced drug delivery. There is evidence that these methods may improve drug efficiency, reduce side effects, and improve patient outcomes. Numerous studies have evaluated the effectiveness of MSCs and membrane-coated nanoparticles for drug delivery [7,11]; results have shown that MSCs were capable of delivering higher concentrations of drugs, and that MCNPs were able to effectively target specific areas and improve the bioavailability of drugs. Furthermore, researches have exposed that the combination of MSCs and membrane-coated nanoparticles increases drug uptake and improves drug efficiency.

Using MSCs and MCNs for enhanced drug delivery has potential, although more study is required to comprehend the exact mechanism of action, efficacy, and safety of this approach [8,20]. More research is required to assess how MSCs and membrane-coated nanoparticles can be used in combination with other drug delivery techniques to improve patient outcomes. MSCs and membrane-coated nanoparticles have the capacity to improve drug delivery. Studies have demonstrated that MSCs can deliver higher concentrations of drugs, while membrane-coated nanoparticles can effectively target specific areas and enhance drug bioavailability [21]. Furthermore, the combination of MSCs and membrane-coated nanoparticles increased drug uptake and improved drug efficiency. Additional researches are required to better understand the exact mechanisms of action, efficacy, and safety of this approach, as well as to evaluate the potential of combining MSCs and MCNPs with other drug delivery techniques to improve patient outcomes. A new drug delivery system utilizing MSCs and membrane-coated nanoparticles has great promise in providing better and more effective treatments for various diseases. The use of these advanced technologies is expected to revolutionize the drug delivery industry and improve patient outcomes [19,22]. As research continues, enhanced drug delivery systems using mesenchymal stem cells and membrane-coated nanoparticles are likely to become even more efficient and effective.

There are two cutting-edge technologies with the potential to revolutionize drug delivery systems: 3D bio printing and ultrasound-based cell therapies. Living tissues, organs, and whole-body parts can be created using 3D bio printing technology; it also has significant potential for creating specialized drug delivery systems that can precisely target the affected area and allow for more effective drugs delivery and treatments. On the other hand, ultrasound-based cell therapies are a new form of targeted drug delivery. This form of drug delivery uses sound waves to target specific cells and delivers drugs or treatments directly to these cells. This technology can advance drug efficacy and decrease the risk of side effects [8].

Both 3D bio printing and ultrasound-based cell therapies are promising technologies that can significantly enhance drug delivery. With 3D bio printing, there is the potential to create delivery systems that can be tailored to the specific needs of each patient. Ultrasound-based cell therapies could improve the accuracy, efficacy, and safety of drug delivery systems and reduce the risk of side effects. Together, these two technologies can revolutionize the delivery of drugs and treatments to patients [17].

Lipid-coated microbubbles and low-intensity pulsed ultrasound (LIPUS) have been demonstrated to enhance chondrogenesis of human mesenchymal stem cells (hMSCs) in 3D printed scaffolds. LIPUS is a noninvasive therapeutic modality that uses low-intensity ultrasonic waves to mediate cellular responses and enhance tissue regeneration. This technique has been widely studied in the context of bone regeneration; however, its application in cartilage regeneration has recently been the focus of several studies.

Lipid-coated microbubbles have been shown to improve the efficacy of LIPUS by targeting ultrasound waves specifically to scaffolds and hMSCs. The microbubbles are composed of a gas-filled lipid shell. When ultrasound waves are applied, the microbubbles oscillate and break up, releasing gas and creating tiny cavitation bubbles. This stimulates hMSCs and induces the formation of cartilage matrix components, thereby enhancing chondrogenesis. In addition, 3D printed scaffolds have been found to be beneficial for hMSCs chondrogenesis [25].

The potential of 3D bioprinted tissue scaffolds to improve bone marrow-derived MSC osteogenesis has been widely studied because these scaffolds provide a 3D structure that can mimic the natural microenvironment of bone tissue [26]. Furthermore, it has been shown that the incorporation of LIPUS stimulation can further enhance the osteogenic potential of MSCs within these scaffolds. LIPUS is a noninvasive technique that has been shown to induce biochemical and mechanobiological changes within the extracellular matrix [27]. It has been demonstrated that these changes are beneficial for the osteogenic differentiation of MSCs. In addition, the scaffolds themselves provide a 3D structure that enables cells to interact and form organized tissues [28]. This increased organization further enhances the osteogenic potential of MSCs. Overall, the combination of 3D bioprinted tissue scaffolds and LIPUS stimulation was found to be a successful strategy for enhancing the osteogenic potential of MSCs. The scaffolds themselves provide an organized structure for cells to interact with and form tissues while LIPUS increases [29].

Nanomedicine and drug delivery systems have revolutionized the approach to medical treatment. Nanomedicine is the medical application of nanotechnology, which involves engineering and manipulating materials at the nanoscale level [30]. This allows for precise delivery of drugs that can be used to target and treat diseases at the cellular and molecular levels [31]. DDSs are designed to increase the effectiveness of medications and reduce their side effects, thereby making them safer and more effective for patients. They can be used to deliver medications to specific areas of the body or enhance the absorption of drugs, allowing them to act faster and more effectively. The development of nanomedicines and DDS is an exciting area of research that has led to major advances in the medical field. They provide novel therapies for a variety of diseases and have the potential to transform the way we approach health care. Nanomedicines and DDSs help improve the safety and efficacy of treatments, which leads to better patient outcomes [31].

## 11. Conclusions

Systemic in vivo use of MSCs is a crucial building block in the development of cell therapy, but there is still much to learn about their pharmacokinetics and pharmacodynamics. There are concerns about potential differences in safety profiles between MSCs derived from various tissues because of the varying biological activities conferred by their sources. There are a variety of options for monitoring inoculation MSCs in vivo, such as the fluorescent nanodiamonds mentioned in this study, and each one has its own set of benefits and drawbacks. The primary goal of future research should be to better understand how specific membrane receptor’s function. As this emerging biomimetic nanotechnology develops, it will be utilised in extensive variety of contexts, eventually maturing until it can satisfy the needs of the clinic. Research into distinct MSCs and how best to utilize them in future treatments will be facilitated by future imaging technologies. It will be required to comprehend the biological variations between MSCs derived from various sources and standardize the growth conditions in order to increase the safety of applying this strategy. Combining MSCs with NPs may be done in two different ways. Initially, anticancer medicines are added to NPs to create MSCs that may be delivered into tumor locations. A gene vector genetically modifies second, drug-loaded NPs to make anticancer proteins, which are then injected into MSCs. In order to deliver two separate medicinal compounds to specific places, this technology functions as a “Trojan Horse.” More research is required to fully understand the role of MSCs in promoting or inhibiting tumor growth, in addition to the difficulties with biocompatibility and advancements in the NP production process. Hence, more research is required before the MSC/NP system may be applied therapeutically in people.

Exosomes made from MSCs can be used instead of MSCs. Exosomes are endogenous nanoparticles that may be manufactured for use in cancer treatment and transport biomolecules. Nevertheless, isolated exosomes have shown shown native MSC anti- or pro-tumor activities. The migration and homing capacities of MSC exosomes require more investigation. In general, more research should be done to understand the interactions between NPs and exosomes in vivo biodistribution as well as their relationships to the tumor microenvironment.

## Figures and Tables

**Table 1:** The role of tumor-promoting factors.

Factors Involved in Tumor Promotion	
IL6, TGF-β1, IL-8	Cytokines
SDF-1, CXCL1, CCL2, CCL5	Chemokines
VEGF, Ang-1, PDGF, IGF	Angiogenic factors
NRG1	Growth factor
periostin, PAI-1, Sema-7A	Other factors
“miR-21-5p, miR-410, MiR-142-3p, miR-23b”	microRNAs

**Table 2:** The literature examining the enhanced drug delivery system using mesenchymal stem cells.

Authors and Year	Results	Reference
Zehui He, et al. (2019)	The drawbacks of traditional therapies and nanosized DDSs are known to be overcome by Cell membrane-coated nanocarriers that actively target tumor locations.	[11]
Xu, et al. (2021)	Engineered exosomes carrying kartogenin (KGN) can effectively promote the chondrogenesis of synovial fluid-derived mesenchymal stem cells (SF-MSCs) both in vivo and in vitro. Exosomes with the MSC-binding peptide E7 shown on their surface are produced by fusing it together with the exosomal membrane protein Lamp 2b, which can target KGN to SF-MSCs, increasing its effective concentration and resulting in stronger chondrogenesis-promoting activity.	[2]
Aoki, et al. (2016)	Biodegradable polymers have been studied for their significant use DDSs and scaffolds for bone regeneration therapy. These polymers are advantageous because of their ability to be rapidly absorbed and replaced with autologous bone. This review covers the performance, features, and types of these polymers in bone regenerative medicine.	[6]
Liao, et al. (2018)	Exosomes are a type of natural nanoscale delivery system that carries signal molecules including proteins and RNAs, and can be used for targeted drug delivery and therapy. This article provides an overview of the characteristics and usages of exosomes, such as their bio functions, biogenesis, purification, isolation, and drug loading, as well as advantages over other nanoparticles and their latent in tumor immunotherapy.	[8]
Barbara, et al. (2019)	Stem cell therapy is being intensively investigated due to its potential as a treatment for treat various diseases. However, many aspects of stem cell therapy have yet to be clarified. While the safety of stem cell applications has been proven, their therapeutic effects are still not spectacular. This review discussed the complex nature of stem cell therapy, its risks, and its potential to be effectively used in regenerative medicine.	[10]
Moku, et al. (2019)	The use of transactivator of transcription (TAT)-functionalized polymeric nanoparticles was investigated to advance the payload capacity of MSCs for small molecule drug delivery. Paclitaxel loaded PLGA nanoparticles were fabricated, TAT was conjugated to the surface and shown to improve intracellular retention and accumulation in MSCs. TAT-conjugation did not change the differentiation and migration capacity of MSCs, and treatment with nanoengineered MSCs significantly slowed the development of tumors.	[13]
Rao, et al. (2022)	MSC-derived exosomes offer tissue repair and regenerative properties similar to those of MSCs and present a viable alternative to the risks of MSC transplantation. This study demonstrated the DDSs and benefits of exosomes and the advancements in MSC exosomes as drug carriers. The concerns and opportunities for using exosomes as DDSs are also discussed.	[17]
Gupta, et al. (2021)	This study demonstrated that MSCs may be used to treat COVID-19 pneumonia. However, MSCs can form clumps in the microenvironment, and the exosomes secreted by them can affect the biological activities of cells. Further research is required to understand the effectiveness of MSCs in treating COVID-19.	[18]

**Table 3:** Biomedical materials and targeted drug delivery using nanoparticles.

Biomedical materials and nanoparticles for targeted drug delivery	
Gold Nanoparticles	Able to carry drugs to targeted cells and tissue
Quantum Dots	Used to track drug delivery and monitor drug release in real time
Liposomes	Used to encapsulate drugs and protect them from degradation
Polymeric Nanoparticles	Used to deliver drugs to specific sites in the body
Carbon Nanotubes	Drug delivery vehicle, H\highly efficient in drug delivery
Dendrimers	Used to improve drug solubility, stability, and bioavailability

## Data Availability

The corresponding author will provide the datasets used for the current study upon reasonable request.
